# From Contrasts to Representations: Rethinking fMRI Approaches for Cortico-Cerebellar Networks

**DOI:** 10.1007/s12311-026-02099-8

**Published:** 2026-10-12

**Authors:** Carolyn N. Irving, Maedbh King, Amanda LeBel

**Affiliations:** 1 Department of Neuroscience, University of California, Berkeley, CA USA; 2 McGovern Institute, Massachusetts Institute of Technology, Cambridge, MA USA

**Keywords:** Cerebellum, fMRI, Connectivity, Encoding models, Cognition

## Abstract

The relationship between the cerebellum and cerebrum remains poorly understood in the neuroimaging literature, despite decades of functional magnetic resonance (fMRI) research. In this opinion article, we argue that this gap reflects fundamental methodological constraints rather than merely a lack of scientific interest. We first discuss challenges in acquiring fMRI data and analysis, with an emphasis on the cerebellum. We then critically evaluate the most common analysis approaches including functional connectivity, univariate contrasts, multivariate pattern analysis, and encoding models, examining the contributions and limitations of each to identify and quantify cortico-cerebellar networks. Through the lens of language processing, we briefly review how these approaches have been applied to characterize cortico-cerebellar networks underlying cognitive function. We conclude by proposing that encoding model frameworks, combined with ecologically valid naturalistic paradigms, offer advantages that can help resolve longstanding debates about cortico-cerebellar network function and organization.

## Introduction

The goal of cognitive neuroscience is to understand how the brain supports cognition. This is often accomplished by relating brain structure and function to behavior. A common method used in the field is functional magnetic resonance imaging (fMRI), which allows us to model the relationship between brain responses and a stimulus or behavior. While the number of fMRI papers has increased exponentially in the past few decades [1], one brain structure is often overlooked: the cerebellum [2]. This is particularly striking given that the cerebellum contains approximately 80% of all neurons in the brain while being only around 10% of total brain volume [3]. Additionally, the cerebellum is implicated in a wide range of clinical disorders and cognitive domains [4, 5] making its systematic exclusion from neuroimaging analyses a significant impediment to understanding whole-brain function [2].

Though the cerebellum has historically been associated with coordination and motor control, anatomical, functional neuroimaging and neuropsychological studies, point to this structure’s role in higher-order cognitive functions [6–8]. The cerebellum is engaged during cognitive tasks including working memory, social cognition, and language [5, 9–12]. Moreover, clinical assessment of individuals with cerebellar disorders also point to higher-order cognitive deficits associated with cerebellar damage [13, 14].

Recent methodological advances in neuroimaging have allowed researchers to study functional and anatomical connectivity between the cerebellum and cerebrum. So far the research has shown that while there is a topographic organization in connectivity between the cerebellum and cerebrum, there is increased individual differences when compared to within cerebrum connectivity [15–18]. Further, researchers have shown that within functional connectivity analyses, the cerebellum is connected to all known cortical networks and shows a bias towards networks involved in higher level cognitive control such as the fronto-parietal network [15]. This has prompted researchers to ask how this connectivity and the variance in connectivity is related to cognitive functioning. For example, cortico-cerebellar connectivity measures are correlated with visual attention and working memory task performance [19–21]. This highlights the importance of studying the cerebellum in network analyses and raises the question: if a goal of fMRI is to map network organization to human behavior, what are we missing by ignoring the cerebellum in these efforts [2]?

Researchers typically overlook the cerebellum in fMRI analyses because it is a difficult brain region to image and requires consideration of cerebellum-specific methodological factors [22–24]. Thus, the choice of fMRI analysis approach can be vital, and different pipelines and modeling strategies offer distinct advantages and disadvantages [25]. Understanding these trade-offs is essential for interpreting existing literature and designing future studies to examine cortico-cerebellar connectivity. Three limitations of past research may constrain our understanding of cortico-cerebellar networks. First, interpreting changes in the magnitude of the Blood-Oxygenation-Level-Dependent (BOLD) signal is not straightforward. Because of the variability and rate of hemodynamics, fMRI connectivity studies do not establish whether regions are anatomically connected or if they interact, only that regions are temporally correlated, co-activate during tasks, or share similar representations. This challenge is compounded when asking both where activity occurs and how regions may interact as a network, since fMRI cannot establish directionality of information flow due to variable, slow hemodynamics. Second, the cerebellum is excluded from many fMRI studies. Over 60% of recent fMRI papers claiming to include whole-brain analyses do not include the cerebellum [2], hindering our ability to characterize cortico-cerebellar networks. Third, standard contrast-based analyses reveal where activity occurs, but not what information cortico-cerebellar regions encode. Knowing whether cerebellar and cortical regions represent similar task-relevant information requires analysis approaches that go beyond contrast-based activation magnitude.

Throughout, we use functional connectivity to refer broadly to the correlated activity, co-activation, and similar representations across spatially distinct brain regions, whether measured via resting-state functional connectivity, task-based analyses, or encoding models, respectively. Functional networks are defined as sets of regions that show consistent functional connectivity with one another. Here, we will discuss cerebellum-specific fMRI concerns, common fMRI analysis approaches, and literature covering cortico-cerebellar connectivity in the language domain. To further our understanding of cortico-cerebellar networks requires measuring, analyzing, and interpreting activity across the whole brain with analysis approaches that can capture finer-grained, task-relevant representations. We propose moving beyond activation magnitude and towards characterizing how cortico-cerebellar regions are tuned to task-relevant stimulus features.

## Methodological Considerations for Imaging the Cerebellum

Cerebellar fMRI faces several challenges. For example, the cerebellum often falls at the edge of the field of view, making it prone to being excluded from image acquisition [2, 24]. Additionally, the anatomical position and dense vasculature surrounding the cerebellum result in a lower signal-to-noise ratio (SNR), the ratio of true signal to background noise, than the cerebral cortex using standard head coils [23, 26]. This adds a confound when performing network studies that compare cerebral and cerebellar regions. In fMRI, the BOLD signal is measured as a proxy of neuronal activity [27] and is sensitive to factors unrelated to cognitive processes, such as respiration and heart rate [28–31]. This may lead to physiological processes being misinterpreted as meaningful signal changes. Some researchers suggest that up to 40% of the BOLD signal recorded from around the cerebellum and brainstem can be explained by heart rate and breathing [24, 32], which is likely due to their position and vasculature. Finally, standard spatial normalization templates often fail for the cerebellum due to its tight, complex folding patterns [22, 33]. This makes it difficult to generate accurate individual-level surfaces and many studies rely on group-level maps that can blur fine-grained spatial information [34]. Using cerebellar specific normalization methods requires additional, separate programs outside of the standard fMRI pipelines. Taken together, these factors can result in lower and less reliable activation estimates and worse model performance compared to cortical regions as well as greater effort from the researchers to include cerebellum in analyses and visualizations.

These issues can be addressed through several approaches. On the data acquisition side, researchers should ensure the field of view includes all cerebellar lobules (see [2] Box 2 for examples). Using multi-band multi-echo sequences improves SNR and reliability of data, particularly for subcortical regions prone to drop-out [35–37]. Collecting more data can improve model performance when SNR is low [38]. Further, normalizing model performance to the noise ceiling, the maximum explainable variance given the noise level of the data, enables more direct comparisons across brain regions because it expresses model performance relative to what is achievable given each region’s noise level. This can be estimated as the correlation between repeated stimulus presentations or Monte Carlo simulations [39–41]. Additionally, the hemodynamic response (HRF) differs across brain region, task, and subject [31, 42–44]. Depending on the experimental design, using finite impulse response (FIR) models rather than canonical boxcar HRF can better account for regional HRF variability because these models make the least assumptions about the shape of the BOLD response [45–48]. Recording physiological information to regress out of the BOLD signal can help reduce the impact of physiological confounds, particularly for the cerebellum [24, 32]. Finally, tools like the Spatially Unbiased Atlas Template (SUIT) can be used to perform spatial normalization while preserving greater anatomical details than typical group spaces (e.g., MNI) [33].

## Strengths and Weaknesses of Common fMRI Analysis Approaches

### Functional Connectivity (FC)

**Fig. 1:**
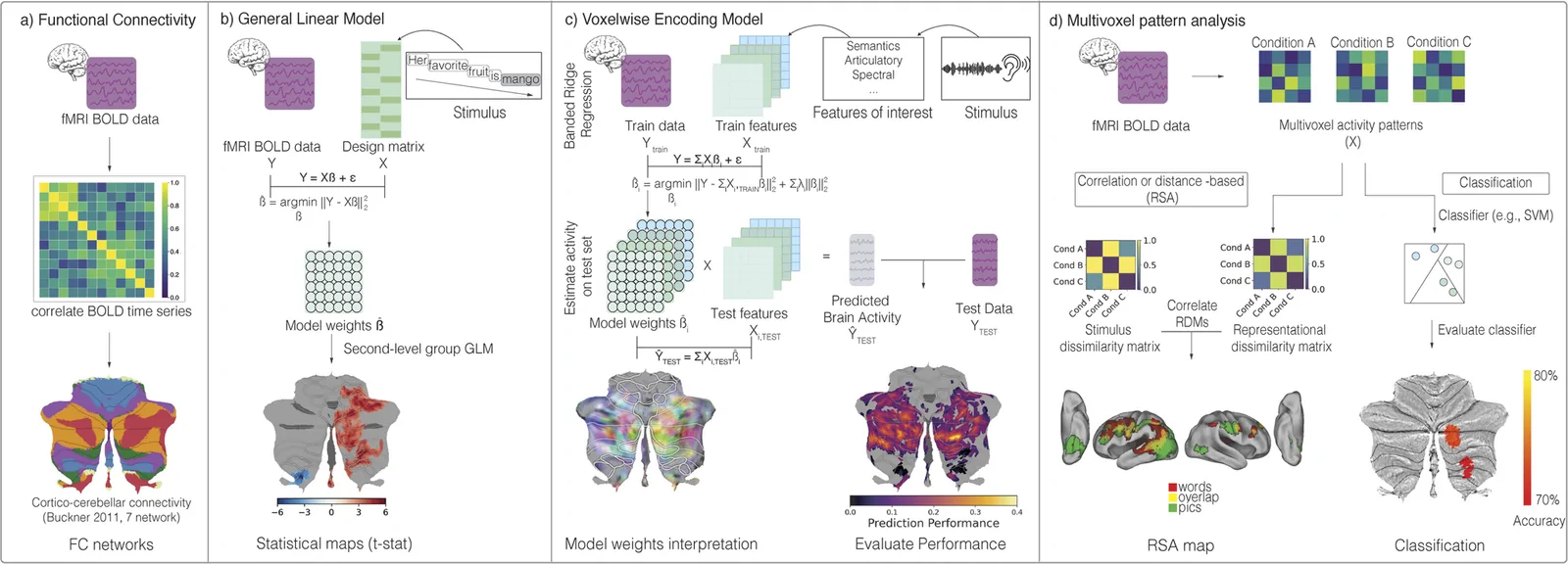
Overview of common fMRI analysis approaches. (**a**) Functional connectivity (FC). BOLD time series are correlated across voxels or ROIs to identify networks with similar fluctuations in activation over time. Cerebellar flatmap shows resting-state 7-network parcellation from [16], figure adapted from [103] (red = default mode network, orange = frontoparietal, purple = ventral attention, green = dorsal attention; blue = somatomotor, white = limbic). (**b**) General Linear Model (GLM). A design matrix of stimulus onsets is used to fit voxelwise activation estimates, which are submitted to a second-level group analysis. Unpublished data (*N* = 21) shown from a semantic prediction task where participants were asked to respond if a sentence was meaningful or meaningless, and the predictability of the target word (shaded grey) was manipulated (*p*<.01 uncorrected, cluster-wise FWE *p*<.05). (**c**) Voxelwise encoding model (VM). Features are extracted from the stimulus then regularized linear regression is used to fit voxelwise models. Models are used to predict responses on a held-out test dataset. Model performance reflects correlation in held-out BOLD data, here with gray regions denoting the model is not significant. Model weights can be interpreted to determine the functional tuning of each voxel. Here, the lexical-semantic weights are plotted with different colors corresponding to selectivity for concepts in semantic space. Data and figures from [10]. (**d**) Multivoxel pattern analysis (MVPA). Voxelwise activity patterns are estimated and extracted for conditions of the task. These multivoxel activity patterns are submitted to either representational similarity analysis (RSA), which compares stimulus and neural dissimilarity matrices, or a classifier trained to distinguish between conditions. Cortical RSA example data shows a semantic representational dissimilarity matrix for words (red), pictures (green), and their overlap (yellow) [104]. Classification example shows voxels in right Crus I and II and Lobule VIII that significantly contributed to discriminating between a language control task and cognitive control task [105]. Color bar indicates accuracy performance

Functional connectivity is an fMRI approach that correlates time series between brain regions [49]. This method is best suited to answer questions that seek to identify networks. A common method is to correlate time series from a seed region of interest to the rest of the brain, though there are various methods [25, 49–52]. This identifies brain regions with similar brain signal changes over time (Fig. 1a). It has been suggested that regions that are temporally coupled at rest also work together during tasks [49].

**Fig. 2:**
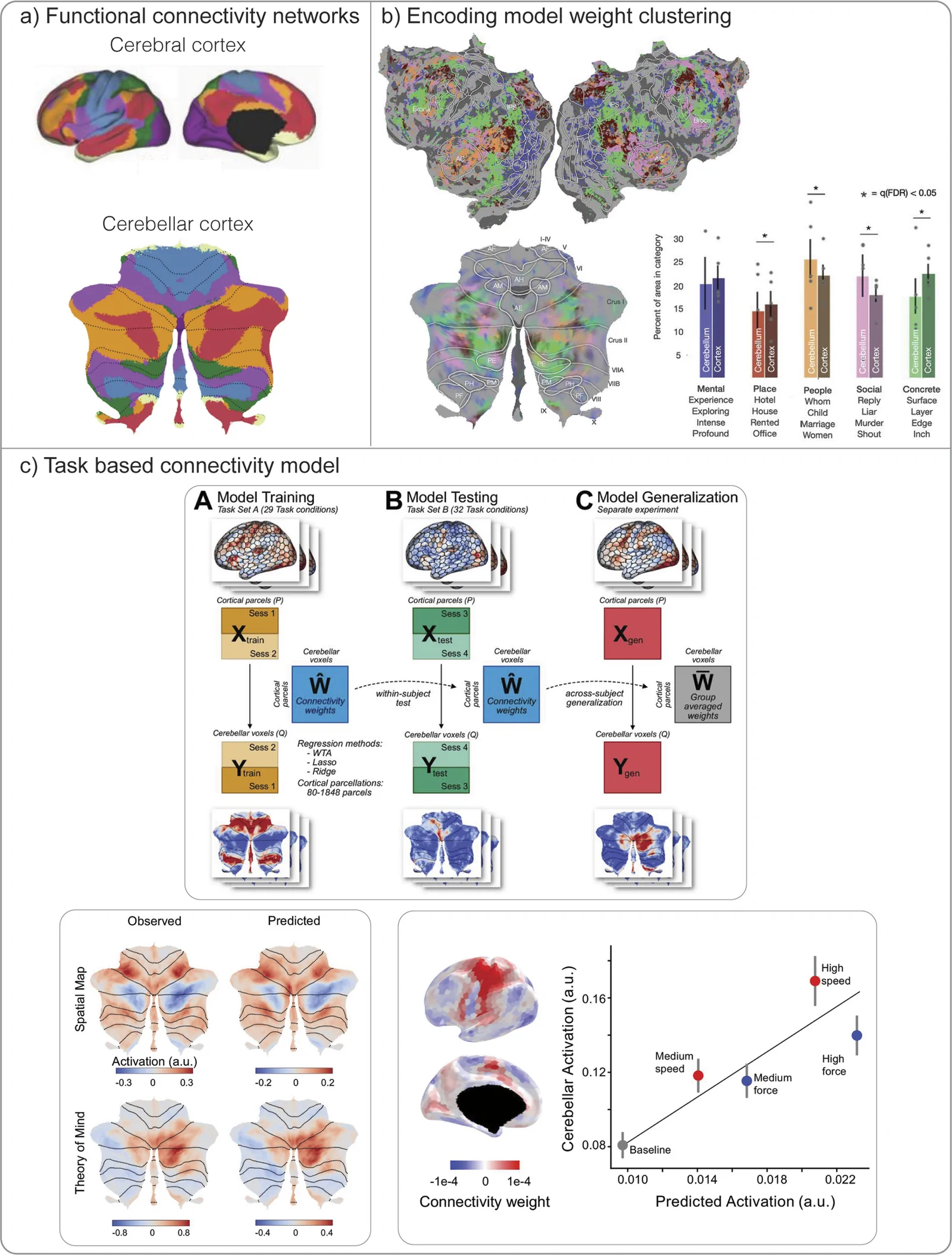
Network connectivity approaches implemented to characterize cortico-cerebellar networks. (**a**) Task free resting-state networks within the cerebellum and the networks that they map to in the cerebral cortex, same color map as Fig. 1a [16, 103]. (**b**) Clustering encoding model weights provide another method to identify networks. Five clusters were identified in the cerebral and cerebellar cortices based on word-level semantic model weights (Figure from [10]). This provides interpretable, high-resolution, and functional defined clusters that can be used to characterize networks. (**c**) Task-based cortico-cerebellar connectivity model. Top panel: task based connectivity model and figure from [69]. A connectivity weight matrix is learned by fitting regression models from cortical parcel task data to predict cerebellar voxel activity. Models are tested on held-out data and tested for generalization. Bottom panel (left): this cortico-cerebellar connectivity model can be applied to generate predicted maps cerebellar activation, based on cortical activation (Figure from [69]). Bottom panel (right): Application of model to predict force and speed task activation from [70]. Deviations above the line are interpreted to show selective engagement of the cerebellum in speed tasks.

Resting state FC has been foundational in characterizing cortico-cerebellar topography (Fig. 2a). These studies have revealed that cerebral networks exist in the cerebellum, with a majority of cerebellar regions mapping to association areas [16, 17]. It has also been shown that there are multiple, overlapping cognitive and emotion networks across the cerebellar cortex [18]. Analysis of cerebellar-cerebral FC patterns has further revealed a gradient of macroscale organization along a primary motor to transmodal processing axis, similar to the cerebral cortex [53]. This finding highlights that there is a domain-specific functional organization across the cerebellar surface that maps to similarly organized cerebral networks. By establishing that the cerebellum, historically considered a primarily motor structure, is part of cognitive networks, this work has laid groundwork for investigating how these networks may work together to support cognitive functioning.

However, factors such as motion, respiration, vasculature, and heart rate can have large influences on the results of FC studies [30, 54]. Furthermore, FC networks are likely confounded by spatial variability in temporal signal-to-noise ratio (tSNR), a measure of the data quality of fMRI time series that quantifies how stable the signal is over time within a voxel or region. This may lead to voxels being assigned to the same network due to highly correlated tSNR [55]. Another limitation is that resting-state FC does not model brain responses to specific stimuli. Without knowing participants’ thoughts during “rest” (e.g., planning their day), it is difficult to distinguish true co-activation from spurious correlations. Task-based FC faces similar challenges. For cerebellar networks, these limitations are magnified by lower tSNR, higher susceptibility to imaging artifacts, and greater physiological noise compared to cortical regions.

One key limitation of these approaches for cortico-cerebellar networks is that network definitions require either a seed region of interest (ROI) or task-based activation to guide parcellation. While these studies have elucidated where co-activation occurs, network boundaries are constrained by prior assumptions rather than derived from the data, which is particularly consequential for the cerebellum, where functional boundaries do not closely follow anatomical boundaries [4, 5, 56]. Even with data-driven parcellations, assigning functional meaning to the resulting networks is not an easy, clear-cut task. Many cerebellar regions support overlapping cognitive functions, making network assignment difficult and potentially leading to mischaracterization of cortico-cerebellar organization.

### General Linear Model (GLM)

The conventional task-based fMRI analysis approach uses a general linear model (GLM) to relate brain activity to experimental conditions [57]. This approach is ideal for localization and mapping. To do this, researchers construct a design matrix with stimulus timing and conditions, then fit a regression model to predict brain activity (Fig. 1b). The GLM output provides voxelwise activation estimates for each condition. A second-level GLM is then fit to test whether activity in a region is associated with a condition across the group [58]. This approach is often referred to as a “mass univariate” analysis and has been foundational in mapping structure-function relationships and remains a popular analysis approach.

There are several methodological limitations of using GLMs in fMRI. First, GLMs typically use ordinary least squares regression, which is prone to overfitting [59], meaning that the model fits the experiment data well but fails to generalize to new data, contributing to reproducibility challenges [60, 61]. Studies rarely include held-out test sets for model validation, making it difficult to assess generalization. Additionally, the GLM relies on a number of assumptions about the data, such as the distribution of errors, that are rarely verified or tested [62]. Particularly for the cerebellum, which has increased noise due to greater susceptibility to artifacts and lower signal quality, these issues of overfitting and assumptions about the data are amplified.

A second limitation with GLMs is that interpretations are restricted to broad category-level contrasts. For example, GLMs are well-suited for contrast analyses since they reveal the location and strength of activation for different conditions [57, 63]. If your research question is about localization alone, a GLM is an appropriate and well powered choice. However, a typical GLM analysis does not provide insight into what information is represented across the brain during a task because GLM models responses to broad experimental conditions [64, 65]. Because GLMs assign a single response estimate to each condition, they cannot distinguish between the many features that could drive activation within a condition. This poses a challenge when trying to compare activation changes in different regions. If a region of the cerebrum and a region in the cerebellum both show strong activation to faces but not houses, does this mean these regions are part of a network? If the goal is to understand what regions respond selectively for faces, the GLM can answer this, providing localization of a face network. If the goal is to understand whether these regions encode similar information about faces, and what features of faces, then the GLM cannot answer this without additional experimental designs and data collection, even when multiple approaches converge on identifying the same activated regions. The GLM approach does not provide a framework for characterizing such network-level organization because it cannot determine whether spatially separate regions encode similar information [66–68]. While activation maps are useful if localization is the goal, we argue that they provide insufficient information about cortico-cerebellar networks and reliance on GLMs alone is an obstacle to characterizing these networks.

In relation to cortico-cerebellar connectivity, one recently proposed application of task-based GLMs is a predictive connectivity model approach. Recent studies have attempted to characterize cerebellar function by training predictive connectivity models on large task batteries [56, 69, 70]. An application of this approach is to identify under which conditions cerebellar activity exceeds cortically predicted baselines, suggesting unique task-specific recruitment of the cerebellum (Fig. 2c). In this approach, a connectivity model is trained to predict cerebellar voxel activity from averaged cortical parcel activity across a wide range of tasks. While this approach has not yet been published working with cognitive paradigms, one example of this approach is within motor learning. Using this approach researchers have found results that suggested greater cerebellar sensitivity to variation in demands on sequence control compared to demands on force control, consistent with findings from patient studies [70]. This result suggests that the cerebellum is more involved in coordinating sequential movements compared to regulating the force of a single movement.

The goal of this connectivity model approach is to quantify functional differences between the cerebellum and cerebrum. However, this framework has some limitations for characterizing cortico-cerebellar networks. First, while there is general agreement that cerebellar BOLD likely reflects local processing [71, 72], this connectivity model makes the additional assumption that those inputs are predominantly cortical in origin [69]. By modeling cerebellar activity as a function of cortical inputs, the approach assumes a cortical-to-cerebellar influence and cannot determine whether cerebellar outputs in turn influence cortical processing, despite anatomical evidence that this is a closed loop pathway [7]. Second, while this analysis may highlight when the cerebellum is selectively recruited, it does not provide information about what this recruitment, or increased activation, represents. Finally, this connectivity model works best with lower-dimensional task-based data and cannot readily handle the higher-dimensional stimuli that enable richer modeling.

### Voxelwise Encoding Models (VM)

Voxelwise encoding models offer a computationally rigorous and flexible approach to modeling fMRI data that addresses several limitations of conventional GLM-based analyses [73, 74]. VM is a type of GLM that incorporates regularization, higher-dimensional features, and generally requires larger datasets. This analysis framework is well-suited to address research questions about what information is represented across the brain, particularly during naturalistic or stimulus rich tasks (e.g., narrative story listening). In this framework, researchers construct feature spaces that reflect hypotheses of interest, such as visual features and semantic embeddings of words in a movie-watching experiment [75, 76]. BOLD activity is then predicted from these features using regularized regression (Fig. 1c).

The VM pipeline follows best practices in data science by splitting data into training and test sets, known as cross-validation. While cross-validation can be implemented in other approaches, it is not standard practice [77, 78]. Models are fit on training data and evaluated on held-out test data, providing quantitative measures of model performance while reducing overfitting and providing a test of generalization. Unlike group-level GLM approaches, VM fits models to participants at the individual level, with each participant serving as an independent replication of the model. This approach allows meaningful individual differences to be quantified [79], which is an advantage for analyzing the cerebellum since there is greater individual variability in the cerebellum than cerebrum. The resulting model weights reveal what information each voxel represents across the cerebral and cerebellar surfaces. This enables the creation of high-dimensional brain maps that characterize brain representations with a finer granularity than GLM activation maps (e.g., Fig. 1b vs. Fig. 1c flatmap results) [10, 75, 80, 81]. Another advantage is that this analysis approach can capture cases where multiple types of information are represented within a single region [82, 83].

The flexibility of VM makes it applicable to any experimental design and particularly well-suited for studying how cortico-cerebellar networks encode complex, naturalistic stimuli. For example, clustering model weights from VM can identify networks based on functional tuning rather than relying on temporal correlation [10, 55] (Fig. 2b). This also moves beyond demonstrating two regions are active during a task (the typical GLM conclusion). For example, by clustering model weights, it is possible to recover networks with precise functional definitions. This approach has revealed that regions within the default mode network are comprised of social and discourse related semantic concepts [55]. This approach has also shown that there are similar conceptual representations across the cerebral and cerebellar cortices [10]. By characterizing both where and what information is represented, VM-derived networks can identify the functional role of cortico-cerebellar networks with a granularity that contrast-based approaches cannot easily achieve. Further, VM-derived networks are based on independent predictions from stimulus features, meaning that shared tuning across regions reflects common representations rather than shared noise, unlike FC networks which share both signal and noise. This offers a data-driven and more interpretable alternative to traditional connectivity analyses for characterizing functional organization across the brain.

Unlike GLM approaches that generally result in wide datasets with many participants and less data per person, VM approaches tend to result in deep datasets with fewer participants and a large amount of data per participant [84, 85]. One weakness of VM is that it requires larger amounts of data to be collected per person, which is not always feasible or necessary if the research question is simply about localization. Another weakness of VM is that it requires an explicit model of the information you want to predict. This can pose challenges when it is unclear what the best features are, like in establishing where somatosensory information is encoded (building a model of somatosensation is less clear than a model of semantics).

### Multivoxel Pattern Analysis (MVPA)

MVPA extends beyond univariate GLM by identifying spatial patterns of activity that discriminate between conditions. While GLM treats each voxel independently (univariate analysis), MVPA models are trained to identify behavior, perceptual, or cognitive brain states based on spatial patterns of activity [86–89] (Fig. 1d). This approach is best utilized to distinguish between experimental conditions or cognitive states. MVPA is a broad category of analyses and commonly uses approaches such as support vector machines, neural networks, and linear discriminant analysis [25, 90–93]. Another common approach, known as “searchlight” mapping, performs multivariate analysis using a spherical multivariate searchlight centered on each voxel [68]. Another multivariate approach is representational similarity analysis (RSA), which compares how similar patterns of brain activity are between stimuli to measure how brain regions represent different information [67].

MVPA offers several advantages. It has greater sensitivity than GLM approaches and can identify voxels that are informative for classifying different task or cognitive states even when there is not a difference in average amplitude of activity across conditions (i.e., not detectable using univariate GLM tests) [94, 95]. Another strength of MVPA is that it can link brain activation to behavior, for instance, when participants make errors, both behavioral accuracy and decoding accuracy decrease together, suggesting a functional role of a brain region [96].

In terms of networks, MVPA can be used as a secondary analysis to identify networks after either establishing resting-state FC networks or t-statistic activation values from a GLM. Few studies performing these analyses include or report on the cerebellum. In one study, MVPA applied to whole-brain resting state FC in individuals with autism spectrum disorder (ASD) revealed clusters of abnormal connectivity in the cerebellum [97]. This finding contributes to the growing evidence implicating cortico-cerebellar connectivity in ASD, highlighting that cerebellar network abnormalities may play a role in ASD, an important component that would have otherwise been missed by cortex-only approaches.

One limitation of MVPA is that it relies on fine-scale spatial patterns to detect subtle distinctions between patterns [66], making classification accuracy affected by fMRI spatial resolution [98]. For the cerebellum specifically, lower SNR, small lobules, and tightly folded cortical sheet mean these limitations are likely worse than in the cerebrum, potentially leading to inaccurate classification accuracy maps. A second, greater, limitation of MVPA is representational ambiguity [94]. MVPA can accurately discriminate between two experimental conditions, but many types of MVPA do not inform us about what sources of information drove successful classification [94, 95, 99, 100]. For example, successful classification of face versus house stimuli could reflect low-level visual features, high-level semantic categories, or motor preparation. This is a significant limitation for the cerebellum, whose contribution to many cognitive functions remains poorly understood. Misattributing classification success to high-level cognitive processing when it may reflect lower-level sensorimotor features risks misleading conclusions about cerebellar function. RSA addresses some of these limitations by comparing representational distances across regions rather than classifying discrete conditions (Fig. 1d). However, RSA is typically conducted at the group level and does not often implement cross-validation or generalization testing, which can limit confidence in these results.

Searchlight MVPA has additional limitations: significant voxels in accuracy maps may not themselves be informative, a few informative voxels can make entire neighborhoods appear significant, and weak information distributed across many voxels can yield successful group classification even when individual voxels are non-informative [101]. This can lead to misleading conclusions because voxels can have significant classifier weights when their average response is the same across conditions [102]. Individual spatial variability can also cause non-overlapping clusters of voxels across participants to be missed at the group level [101], and generalizability can be limited by classifier choice and varying parameters [25].

## Cortico-Cerebellar Connectivity Approaches to Characterize Whole-Brain Language Networks

With these methodological considerations in mind, we can evaluate what these approaches have revealed, and leave unanswered, about cortico-cerebellar networks in the language domain. Since the late 1980s, there has been an ongoing debate about the role of the cerebellum in language processing [106, 107]. This has led to a rise of papers investigating whether the cerebellum plays a core role in the language network. These approaches have established a role of the cerebellum in various domains of language processing from phonological processing and verb generation to sentence comprehension and narrative story listening [4, 5, 10, 11, 108–111]. Many of these cerebellar regions that respond in language tasks are also functionally connected to cortical language regions [18, 110]. Together, recent studies demonstrate that the cerebellum is a likely part of the core language network. However, how the cerebellum contributes to language processing and the language network remains unclear.

### Internal Models Framework and Predictions

One theory of how the cerebellum contributes to language processing is through predictive linguistic processing (e.g., anticipating upcoming words in a sentence given the current context), an extension of the internal model framework originally conceptualized in the motor domain [112–114]. This hypothesis makes several predictions about cortico-cerebellar network interactions. It predicts a specific directional information flow such that the cerebral cortex sends signals to the cerebellum, while the cerebellum sends predictive signals back to the cerebrum. It also predicts dynamic coupling changes with strong cortico-cerebellar coupling that shifts as processing becomes more automated.

There are inherent limitations of fMRI that make it difficult to test these predictions fully, regardless of analysis approach. First, fMRI connectivity can reveal that cortical language regions and cerebellar lobules VI and VII are functionally coupled but cannot establish the directionality of this coupling or whether cerebellar activity reflects predictive versus error signals, a key distinction for internal models. Given the BOLD signal reflects the hemodynamic response that occurs over seconds [115] it is difficult to capture real-time prediction-error signals, and other methods such as magnetoencephalography (MEG) would be more appropriate with its fine temporal resolution. Second, the BOLD signal is a proxy for neuronal activity, most likely reflecting synaptic inputs and local processing, rather than output of firing activity [71, 116]. This makes it difficult to interpret whether cerebellar BOLD activation reflects incoming information or outgoing predictions, a key part of internal models. Third, because the HRF differs between brain regions, establishing which region was active first is not straightforward due to the potential differences in hemodynamic coupling between regions, and variability across individuals [44, 117]. Thus, testing if cortico-cerebellar coupling reflects information being sent to the cerebellum or vice versa is not straightforward. Conclusions about the directionality of cortico-cerebellar information flow should be made with caution regardless of which fMRI analysis approach is used.

### Cortico-Cerebellar Networks in Language Processing

In the language domain, resting-state FC studies have identified that cortical language regions are functionally connected with cerebellar lobules VI and VII [18]. This analysis approach has also revealed that cortico-cerebellar FC is altered in chronic stroke patients with aphasia [118]. Moreover, decreased FC between cerebellar seed regions and left cortical language regions was related to performance on a naming task, suggesting an important role of cortico-cerebellar networks following cortical strokes. This result could also be explained by crossed cerebellar diaschisis, with diminished input via corticopontocerebellar pathways due to cerebral damage [108, 119]. A remaining question from these studies is what role the cerebellum may be playing in this language network: are these FC networks related to syntax, semantic processing, or another component of language? A limitation in these studies is the ambiguity of networks. Seed-based approaches require assuming which cerebellar region belongs to the language network before the analysis begins. Even in data-driven parcellations, assigning a functional label to an identified network requires interpretation, often from GLM contrasts. Given that the same cerebellar regions respond to different aspects of language, that interpretation is not straightforward [111].

A recent paper characterized cerebellar contributions to the language network using a GLM based approach [110]. In this study, they use a functional localizer to identify language-responsive regions by contrasting meaningful versus meaningless sentences, across written and auditory modalities [120]. Individual functional ROIs were then defined as the top 10% most responsive voxels within the group-level parcel. This revealed four regions in the right posterior cerebellum: (1) lateral VI, (2) posterior VI and Crus I/II, (3) Crus I, II and lobule VIIb, (4) lobule VIIb and VIIIa. They then examined how these regions respond to other stimuli testing across modalities and for selectivity across task domains. Of these four regions, only one, which is localized to CrusI/II and Lobule VIIb, was found to be selective for language over non-linguistic tasks. This means that within this ROI, the greatest percent BOLD signal change was found for the condition of sentences vs. nonwords over other tasks they tested, such as articulation, finger tapping, spatial working memory, and more. This pattern of activation behavior is similar to core language regions in the cortex, such that cortical regions identified using a functional localizer also show selectivity for language (sentences vs. nonwords) over other cognitive tasks. Additionally, these cerebellar regions are functionally connected to cerebral language regions. Taken together, this localization and FC approach suggests that cerebral language regions and cerebellar regions, particularly CrusI/II and Lobule VIIb, form a core language network. Similarly, a large-scale meta-analysis found selective and overlapping regions of cortical and cerebellar activated for various domains of language processing, also pointing to a larger language network that includes cerebellar regions [111]. An open question that remains is what drives each network region’s activation? Whether the same linguistic features are encoded in this cortico-cerebellar language selective network cannot be determined from activation magnitude alone.

Few MVPA studies have examined cortico-cerebellar language networks. One recent paper sought to decode activity patterns in the cerebral and cerebellar cortices during bilingual language processing [121]. Using a cross-subject temporal decoding approach, a classifier was trained to identify which timepoint of a narrative a participant was at, based on pattern similarity across individuals. Decoding accuracy served as a measure of how consistent a feature was across participants, which the authors term “informativeness”. They report that cortico-cerebellar connectivity shows greater informativeness than intra-cerebellar connectivity alone, claiming that the cerebellum primarily represents language through cortico-cerebellar interactions. However, a key limitation is that SNR and reliability differ substantially between cerebellar and cortical regions, and this was neither controlled for nor reported. Without accounting for these differences, it is difficult to determine whether improved decoding accuracy reflects richer cortico-cerebellar representations or simply a stronger signal in cortical regions.

Encoding models have been used to show that there are distinct semantic networks across the cerebral and cerebellar cortex [10, 55, 79–81]. Unlike localizer-based approaches that may explicitly control for non-linguistic factors such as executive demands, encoding models do not isolate language comprehension from other cognitive processes. While this could be a disadvantage, it allows naturalistic language representations to be mapped across the brain without being constrained by experimenter-defined contrasts. In one study exploring semantic networks between the cortex and cerebellum, participants listened to narrative stories [10]. Using VM, semantic features across the language hierarchy were used to predict BOLD activity. This revealed that there was widespread, conceptual semantic information represented across the cerebellar surface, similar to the cortical surface. Further, clustering of the encoding model weights showed distinct semantic networks (e.g., social concepts) across both cerebral and cerebellar cortices which could indicate shared conceptual networks (Fig. 2b). Additionally, the cerebellar cortex showed a bias towards the social network compared to the other semantic networks. This demonstrates the feasibility of using encoding models to identify interpretable, fine-grained, functional networks that do not require a priori ROI selection, group-level averaging, or smoothing.

## Concluding Remarks and Future Directions

The role of the cerebellum in cognition is an increasingly studied topic, though how the cerebellum supports cognition remains enigmatic [122]. Better understanding cortico-cerebellar connectivity and networks can elucidate cerebellar contributions across domains, yet there are methodological constraints that limit progress in analyzing and interpreting these networks.

The cerebellum is a difficult region of the brain to image and requires considerations in MR sequences as well as extra steps after data collection, like separate normalization and visualization. Increased susceptibility to multiple forms of artifacts results in greater noise in the cerebellar BOLD signal, which should be considered before comparing cerebellar and cerebral regions (e.g., noise ceiling correction). The high rate of cerebellar exclusion in studies, particularly those claiming to be whole-brain, further interferes with progress in identifying and understanding cortico-cerebellar networks.

Beyond imaging-specific challenges, the inherent limitations of the fMRI BOLD signal constrain what conclusions can be drawn about cortico-cerebellar networks regardless of analysis approach. fMRI cannot currently establish directionality or interactions between brain regions. When the research question concerns how cerebellar and cortical regions integrate information, other methods may be more appropriate. For example, MEG can detect rapid cerebellar responses and can demonstrate how cerebellar and cortical regions are differentially involved in task processing with a temporal resolution fMRI cannot achieve [123–125]. Claims about cortico-cerebellar interactions should therefore be made with caution and kept within the limitations of what fMRI can establish.

Common fMRI analysis approaches leave open questions about how cortico-cerebellar networks support cognition. When multiple approaches converge on the same result, for example multiple fMRI analysis approaches point to the cerebellum as a part of the core language network, this agreement can build confidence in a finding. However, there are cases where univariate and multivariate approaches provide differing results because they are sensitive to different sources of variance in the data [95]. This highlights the importance of selecting an analysis approach matched to the research question. Connectivity analyses to date reveal when the cerebrum and cerebellum are active or functionally coupled but provide limited insight into what information these regions encode. This motivates analysis approaches that can characterize cortico-cerebellar networks based on task-related representations.

We propose that encoding models offer a promising path forward for cortico-cerebellar networks. By predicting BOLD activity from explicitly defined stimulus features, VM produces interpretable, fine-grained maps of what information each region encodes across cerebral and cerebellar surfaces. Because predictions are derived independently from stimulus features rather than from correlated time series, shared encoding model weights across regions more likely reflect common representations than shared noise, a problem that confounds FC-based networks. Clustering model weights can recover functional networks of voxels that share functional tuning based on shared representations. This framework offers a more principled approach for defining cortico-cerebellar networks.

Many have advocated for using more complex naturalistic stimuli during fMRI experiments [126, 127]. Given that real-world skills and exploration involve a rich and dynamic interaction with our environments, it is beneficial to study the brain using more ecologically valid designs. While the typical fMRI analysis can only handle a few conditions, VM employs ridge regression allowing for a large number stimulus features to be fit at once [83]. Together, using naturalistic stimuli with encoding model approaches can help characterize the shared and distinct information that the cerebrum and cerebellum represent, which remains an open question across domains.

Future work could extend this approach in several directions. Applying encoding models during story listening or moving watching, performing clustering on the model weights to identify networks, and then comparing networks with diffusion-based structural connectivity would test whether functionally defined cortico-cerebellar networks align with anatomical pathways [128]. Longitudinal studies could further examine how cortico-cerebellar networks change during skill learning. Addressing these methodological gaps will advance toward a more complete understanding of how the cerebrum and cerebellum work together to support cognition.

## Data Availability

No datasets were generated or analysed during the current study.
